# Child-Parent Relation and Older Adults' Health: A Cross-Cultural Comparison Between China and the United States

**DOI:** 10.1093/geroni/igab046.1521

**Published:** 2021-12-17

**Authors:** Peiyi Lu, Dexia Kong, Mack Shelley

**Affiliations:** 1 Columbia University, Fort Lee, New Jersey, United States; 2 The Chinese University of Hong Kong, Hong Kong , Hong Kong; 3 Iowa State University, Ames, Iowa, United States

## Abstract

Western culture emphasizes independence in the child-parent relationship while Chinese culture values interdependence between adult children and older parents. This study compared the association of child-parent relationships with older adults’ multidimensional health over time in the U.S. and China. Two waves of data (2012-2015) from HRS and CHARLS were used (n=6,641, aged ≥65). Linear regression models were estimated. Results showed that, compared to Chinese older adults, fewer older Americans co-resided with or lived nearby their children, had less weekly contact, and fewer financial transfers from/to their children. Most child-parent relationship variables were nonsignificant predictors of older Americans’ health. However, a closer child-parent relationship was linked to fewer depressive symptoms and better cognition among older Chinese. Co-residence was associated with poorer health among Chinese parents. The associations of child-parent relationships with older adults’ health exhibited cross-cultural differences. A cultural perspective is recommended in understanding how family relations affect older adults’ health.

